# Modelling and Recognition of Protein Contact Networks by Multiple Kernel Learning and Dissimilarity Representations

**DOI:** 10.3390/e22070794

**Published:** 2020-07-21

**Authors:** Alessio Martino, Enrico De Santis, Alessandro Giuliani, Antonello Rizzi

**Affiliations:** 1 Department of Information Engineering, Electronics and Telecommunications, University of Rome “La Sapienza”, Via Eudossiana 18, 00184 Rome, Italy; enrico.desantis@uniroma1.it (E.D.S.); antonello.rizzi@uniroma1.it (A.R.); 2 Department of Environment and Health, Istituto Superiore di Sanità, Viale Regina Elena 299, 00161 Rome, Italy; alessandro.giuliani@iss.it

**Keywords:** dissimilarity spaces, support vector machines, kernel methods, computational biology, systems biology, protein contact networks

## Abstract

Multiple kernel learning is a paradigm which employs a properly constructed chain of kernel functions able to simultaneously analyse different data or different representations of the same data. In this paper, we propose an hybrid classification system based on a linear combination of multiple kernels defined over multiple dissimilarity spaces. The core of the training procedure is the joint optimisation of kernel weights and representatives selection in the dissimilarity spaces. This equips the system with a two-fold knowledge discovery phase: by analysing the weights, it is possible to check which representations are more suitable for solving the classification problem, whereas the pivotal patterns selected as representatives can give further insights on the modelled system, possibly with the help of field-experts. The proposed classification system is tested on real proteomic data in order to predict proteins’ functional role starting from their folded structure: specifically, a set of eight representations are drawn from the graph-based protein folded description. The proposed multiple kernel-based system has also been benchmarked against a clustering-based classification system also able to exploit multiple dissimilarities simultaneously. Computational results show remarkable classification capabilities and the knowledge discovery analysis is in line with current biological knowledge, suggesting the reliability of the proposed system.

## 1. Introduction

Dealing with structured data is an evergreen challenge in pattern recognition and machine learning. Indeed, many real-world systems can effectively be described by structured domains such as networks (e.g., images [1,2]) or sequences (e.g., signatures [3]). Biology is a seminal field in which many complex systems can be described by networks [4], as the biologically relevant information resides in the interaction among constituting elements: common examples include protein contact networks [5,6], metabolic networks [7] and protein–protein interaction networks [8,9].

Pattern recognition in structured domains poses additional challenges as many structured domains are non-metric in nature (namely, the pairwise dissimilarities in such domains might not satisfy the four properties of a metric: non-negativity, symmetry, identity, triangle inequality) and patterns may lack any geometrical interpretation [10].

In order to deal with such domains, five mainstream approaches can be pursued [10]:

1. Feature generation and/or feature engineering, where numerical features are extracted ad-hoc from structured patterns (e.g., using their properties or via measurements) and can be further merged according to different strategies (e.g., in a multi-modal way [11]);
2. Ad-hoc dissimilarities in the input space, where custom dissimilarity measures are designed in order to process structured patterns directly in the input domain without moving towards Euclidean (or metric) spaces. Common—possibly parametric—edit distances include the Levenshtein distance [12] for sequence domains and graph edit distances [13] for graphs domains;
3. Embedding via information granulation and granular computing [3,14,15,16,17,18,19,20,21,22,23,24,25];
4. Dissimilarity representations [26,27,28], where structured patterns are embedded in the Euclidean space according to their pairwise dissimilarities;
5. Kernel methods, where the mapping between the original input space and the Euclidean space exploits positive-definite kernel functions [29,30,31,32,33].

This paper proposes a novel classification system based on an hybridisation of the latter two strategies: while dissimilarity representations see the (structured) patterns according to the pairwise dissimilarities, kernel methods encode pairwise similarities. Nonetheless, the class of properly-defined kernel functions is restricted: the (conditionally) positive definitiveness may not hold in case of non-metric (dis)similarities. The use of kernel methods in state-of-the-art (non-linear) classifiers such as Support Vector Machines (SVM) [34,35] is strictly related to their (conditionally) positive definitiveness due to the quadratic programming optimisation involved: indeed, non-(conditionally) positive definite kernels do not guarantee convergence to the global optimum. Although there is some research about learning from indefinite kernels (see, e.g., [36,37,38,39,40]), their evaluation on the top of Euclidean spaces (e.g., dissimilarity spaces) retain the (conditionally) positive definitiveness, devoting matrix regularisation or other tricks to foster positive definitiveness.

The proposed classification system is able to simultaneously explore multiple dissimilarities following a multiple kernel learning approach, where each kernel considers a different (dissimilarity) representation. The relative importance of the several kernels involved is automatically determined via genetic optimisation in order to maximise the classifier performance. Further, the very same genetic optimisation is in charge of determining a suitable subset of representative (prototypes) patterns in the dissimilarity space [27] in order to shrink the modelling complexity. Hence, the proposed system allows a two-fold a posteriori knowledge discovery phase:

1. By analysing the kernel weights, one can determine the most suitable representation(s) for the problem at hand;
2. The patterns elected as representatives for the dissimilarity space (hence determined as pivotal for tracking the decision boundary amongst the problem-related classes) can give some further insights for the problem at hand.

In order to validate the proposed classification system, a bioinformatics-related application is considered, namely protein function prediction. Proteins’ 3D structure (both tertiary and quaternary) can effectively be modelled by a network, namely the so-called Protein Contact Network (PCN) [5]. A PCN is a minimalistic (unweighted and undirected) graph-based protein representation where nodes correspond to amino-acids and edges between two nodes exist whether the Euclidean distance between residues’ α-carbon atom coordinates is within [4,8]Å. The lower bound is defined in order to discard trivial connections due to closeness along the backbone (first-order neighbour contacts), whereas the upper bound is defined by considering the peptide bonds geometry (indeed, 8Å roughly correspond to two van der Waals radii between residues’ α-carbon atoms [41]). It is worth stressing that both nodes labels (i.e., the type of amino-acid) and edges labels (i.e., the distance between neighbour residues) are deliberately discarded in order to focus only on proteins’ topological configuration. Despite the minimalistic representation, PCNs have been successfully used in pattern recognition problems for tasks such as solubility prediction/folding propensity [42,43] and physiological role prediction [44,45,46]; furthermore, their structural and dynamical properties have been extensively studied in works such as [47,48,49,50].

In order to investigate how the protein function is related to its topological structure, a subset of the entire Escherichia coli bacterium proteome, correspondent to E. coli proteins whose 3D structure is known, is considered. The problem itself is cast into a supervised pattern recognition task, where each pattern (protein) is described according to eight different representations drawn by its PCN and its respective Enzyme Commission (EC) number [51] that serves as the ground-truth class label. The EC nomenclature scheme classifies enzymes according to the chemical reaction they catalyse and a generic entry is composed by four numbers separated by periods. The first digit (1–6) indicates one of the six major enzymatic groups (EC 1: oxidoreductases; EC 2: transferases; EC 3: hydrolases; EC 4: lyases; EC 5: isomerases; EC 6: ligases) and the latter three numbers represent a progressively finer functional enzyme classification. In this work, only the first number is considered. However, proteins with no enzymatic characteristics (or proteins for which enzymatic characteristics are still unknown nowadays) are not provided with an EC number, thus an additional class of not-enzymes will be considered, identified by the categorical label 7. It is worth noting that the EC classification only loosely relates to global protein 3D configuration, given that structure is affected by many determinants other than catalysed reactions like solubility, localisation in the cell, interaction with other proteins and so forth. This makes the classification task intrinsically very difficult.

This paper is organised as follows: Section 2 overviews some theory related to kernel methods and dissimilarity spaces; Section 3 presents the proposed methodology; Section 4 shows the results obtained with the proposed approach, along with a comparison against a clustering-based classifier (also able to explore multiple dissimilarities), and we also provide some remarks on the two-fold knowledge discovery phase. Finally, Section 5 concludes the paper. The paper also features two appendices: Appendix A describes in detail the several representations used for describing PCNs, whereas Appendix B lists the proteins selected as prototypes for the dissimilarity representations.

## 2. Theoretical Background

Let $D=\{x_{1},\ldots ,x_{N_{P}}\}$ be the dataset at hand lying in a given input space X. Moving the problem towards a dissimilarity space [26] consists in expressing each pattern from D according to the pairwise distances with respect to all other patterns, including itself. In other words, the dataset is cast into the pairwise distance matrix $D\in R^{N_{P}\times N_{P}}$ defined as:

(1) $$D_{i,j}=d\left(x_{i},x_{j}\right)\;\forall i,j=1,\ldots ,N_{P}\;,$$

where d(·,·) is a suitable dissimilarity measure in D, that is d:D×D→R. Without loss of generality, hereinafter let us consider D to be symmetric: if d(·,·) is at least symmetric, D is trivially symmetric; in case of asymmetric dissimilarity measures, D can be ‘forced’ to be symmetric, e.g., $D:=\frac{1}{2}\left(D+D^{T}\right)$. The major advantage in moving the problem from a generic input space X towards $R^{N_{P}\times N_{P}}$ is that the latter can be equipped with algebraic structures such as the inner product or the Minkowski distance, whereas the former might not be metric altogether. As such, in the latter, standard computational intelligence and machine learning techniques can be used without alterations [10]. On the negative side, the explicit evaluation of D can be computationally expensive as it leads to a time and space complexity of $O(N_{P}^{2})$. To this end, in [27], a ‘reduced’ dissimilarity space representation is proposed, where a subset of prototype patterns R⊂D is properly chosen and each pattern is described according to the pairwise distances with respect to the prototypes only. This leads to the definition of a ‘reduced’ pairwise distance matrix $\bar{D}\in R^{N_{P}\times \left|R\right|}$ defined as:

(2) $${\bar{D}}_{i,j}=d\left(x_{i},x_{j}\right)\;\forall i=1,\ldots ,N_{P},\;\forall j=1,\ldots ,\left|R\right|.$$

Since usually |R|<|D|, there is no need to solve a quadratic complexity problem such as evaluating Equation (1). On the negative side, however, the selection of the subset R is a delicate and challenging task [10] since:

1. They must well-characterize the decision boundary between patterns in the input space;
2. The fewer, the better: the number of representatives has a major impact on the model complexity (cf. Equation (1) vs. Equation (2)).

Several heuristics have been proposed in the literature, ranging from clustering the input space to (possibly class-aware) random selection [10,27,52].

Kernel methods are usually employed whether the input space has an underlying Euclidean geometry. Indeed, the simplest kernel (namely, the linear kernel [30,53]) is the plain inner product between real-valued vectors. The kernel matrix K (also known as the Gram matrix) can easily be defined as:

(3) $$K_{i,j}=\langle x_{i},x_{j}\rangle \;\forall i,j=1,\ldots ,N_{P}.$$

Let *K* be a symmetric and positive semi-definite kernel function from the input space X towards R, that is K:X×X→R such that

(4) $$\begin{array}{cc} K\left(x_{i},x_{j}\right)=K\left(x_{j},x_{i}\right)\; & \forall x_{i},x_{j}\in X \end{array}$$

(5) $$\begin{array}{cc} \sum_{i=1}^{N_{P}}\sum_{j=1}^{N_{P}}c_{i}c_{j}K\left(x_{i},x_{j}\right)\geq 0\; & \forall c_{i},c_{j}\in R,\forall x_{i},x_{j}\in X. \end{array}$$

As in the linear kernel case, starting from pairwise kernel evaluations, one can easily evaluate the kernel matrix as

(6) $$K_{i,j}=K\left(x_{i},x_{j}\right)\;\forall i,j=1,\ldots ,N_{P}$$

and if K is a positive semi-definite kernel matrix, then *K* is a positive semi-definite kernel function. One of the most intriguing kernel methods property relies on the so-called kernel trick [29,30]: kernel of the form Equations (4) and (5) are also known as Mercer’s kernel as they satisfy the Mercer condition [32]. Such kernel functions can be seen as the inner product evaluation on a high-dimensional (or possibly infinite-dimensional) and usually unknown Hilbert space H. The kernel trick is usually described by the following, seminal, equation:

(7) $$K\left(x,y\right)={\langle \phi \left(x\right),\phi \left(y\right)\rangle }_{H},$$

where ϕ:X→H is the implicit (and usually unknown) mapping function. The need for using a non-linear and higher-dimensional mapping is a direct consequence of Cover’s theorem [33]. Thanks to the kernel trick, one can use one of the many kernel functions available (e.g., polynomial, Gaussian, radial basis function) in order to perform such non-linear and higher-dimensional mapping without knowing and explicitly evaluating the mapping function ϕ(·). Further, kernel methods can be used in many state-of-the-art classifiers such as (kernelised) SVM [35,54].

In multiple kernel learning, the kernel matrix K is defined as a properly-defined combination of a given number of $N_{K}$ kernels. The most intuitive combination is a linear combination of the form:

(8) $$K=\sum_{i=1}^{N_{K}}\beta_{i}K^{\left(i\right)},$$

where sub-kernels $K^{\left(i\right)}$ are single Mercer’s kernels. The weights $\beta_{i}$ can be learned according to different strategies and can be constrained in several ways—see, e.g., [55,56,57,58,59,60,61], or the survey [62]. The rationale behind using a multiple kernel learning with respect to a plain single kernel learning depends on the application: for example, if data come from different sources, one might want to explore such different sources according to several kernels or, dually, one might want to explore the same data using different kernels, where such different kernels may differ in shape and/or type. In this work, a mixture between the two approaches is pursued: same source (PCN), but different representations (see Appendix A). Further, a linear convex combination of radial basis function kernels is employed. The *i*th radial basis function kernel is defined as

(9) $$K_{j,k}^{\left(i\right)}=\exp\left\{-\gamma_{i}\cdot {∥x_{j}-x_{k}∥}^{2}\right\}\;\forall j,k=1,\ldots ,N_{P}$$

and $\gamma_{i}$ is its shape parameter. Further, the weights $\beta_{i}$ are constrained as

(10) $$\begin{array}{cc} \sum_{i=1}^{N_{K}}\beta_{i}=1 & \; \end{array}$$

(11) $$\begin{array}{cc} \beta_{i}\in \left[0,1\right]\; & \mathrm{for}\;i=1,\ldots ,N_{K}. \end{array}$$

It is rather easy to demonstrate that these selections for both kernels and weights lead to the final kernel matrix (as in Equation (8)) which still is a valid Mercer’s kernel, therefore it can be used on kernelised SVMs. Indeed, Cristianini and Shawe-Taylor in [31] showed that the summation of two valid kernels is still a valid kernel. Further, Horn and Johnson in [63] showed that a positive semi-definite matrix multiplied by a non-negative scalar is still a positive semi-definite matrix. Merging these two results automatically prove that kernels of the form (8) and (9) with constraints (10) and (11) are valid kernels.

## 3. Proposed Methodology

Let D be the dataset at hand, split into three non-overlapping subsets $D_{\mathrm{TR}}$, $D_{\mathrm{VAL}}$ and $D_{\mathrm{TS}}$ (namely training set, validation set and test set). Especially for structured data, several representations (e.g., set of descriptors) might hold for the same data, therefore let $\left\{X^{\left(1\right)},\ldots ,X^{\left(N_{R}\right)}\right\}$ be the set of $N_{R}$ representations, split in the same fashion (i.e., ${\left\{X_{\mathrm{TR}}^{\left(i\right)}\right\}}_{i=1}^{N_{R}}$, ${\left\{X_{\mathrm{VAL}}^{\left(i\right)}\right\}}_{i=1}^{N_{R}}$ and ${\left\{X_{\mathrm{TS}}^{\left(i\right)}\right\}}_{i=1}^{N_{R}}$). Finally, let $\left\{d^{\left(1\right)}\left(\cdot ,\cdot \right),\ldots ,d^{\left(N_{R}\right)}\left(\cdot ,\cdot \right)\right\}$ be the set of dissimilarity measures suitable for working in their respective representations.

The respective training, validation and test pairwise dissimilarity matrices, as in Equation (1) can be evaluated as follows:

(12) $$\begin{array}{cccccc} \; & D_{\mathrm{TR}}^{\left(1\right)}=d^{\left(1\right)}\left(X_{\mathrm{TR}}^{\left(1\right)},X_{\mathrm{TR}}^{\left(1\right)}\right) & \; & \;\ldots & \; & \;D_{\mathrm{TR}}^{\left(N_{R}\right)}=d^{\left(N_{R}\right)}\left(X_{\mathrm{TR}}^{\left(N_{R}\right)},X_{\mathrm{TR}}^{\left(N_{R}\right)}\right)\\ \; & D_{\mathrm{VAL}}^{\left(1\right)}=d^{\left(1\right)}\left(X_{\mathrm{VAL}}^{\left(1\right)},X_{\mathrm{TR}}^{\left(1\right)}\right) & \; & \;\ldots & \; & \;D_{\mathrm{VAL}}^{\left(N_{R}\right)}=d^{\left(N_{R}\right)}\left(X_{\mathrm{VAL}}^{\left(N_{R}\right)},X_{\mathrm{TR}}^{\left(N_{R}\right)}\right)\\ \; & D_{\mathrm{TS}}^{\left(1\right)}=d^{\left(1\right)}\left(X_{\mathrm{TS}}^{\left(1\right)},X_{\mathrm{TR}}^{(1}\right) & \; & \;\ldots & \; & \;D_{\mathrm{TS}}^{\left(N_{R}\right)}=d^{\left(N_{R}\right)}\left(X_{\mathrm{TS}}^{\left(N_{R}\right)},X_{\mathrm{TR}}^{\left(N_{R}\right)}\right). \end{array}$$

Let $w\in {\left\{0,1\right\}}^{|D_{\mathrm{TR}}|}$ be a binary vector in charge of selecting columns from all matrices in Equation (12): the full pairwise dissimilarities can be sliced to their ‘reduced’ versions (cf. Equation (1) vs. Equation (2)), hence:

(13) $$\begin{array}{ccc} {\bar{D}}_{\mathrm{TR}}^{\left(1\right)}=D_{\mathrm{TR}}^{\left(1\right)}\left(:\;,w\right) & \;\ldots & \;{\bar{D}}_{\mathrm{TR}}^{\left(N_{R}\right)}=D_{\mathrm{TR}}^{\left(N_{R}\right)}\left(:\;,w\right)\\ {\bar{D}}_{\mathrm{VAL}}^{\left(1\right)}=D_{\mathrm{VAL}}^{\left(1\right)}\left(:\;,w\right) & \;\ldots & \;{\bar{D}}_{\mathrm{VAL}}^{\left(N_{R}\right)}=D_{\mathrm{VAL}}^{\left(N_{R}\right)}\left(:\;,w\right)\\ {\bar{D}}_{\mathrm{TS}}^{\left(1\right)}=D_{\mathrm{TS}}^{\left(1\right)}\left(:\;,w\right) & \;\ldots & \;{\bar{D}}_{\mathrm{TS}}^{\left(N_{R}\right)}=D_{\mathrm{TS}}^{\left(N_{R}\right)}\left(:\;,w\right). \end{array}$$

where, due to the number of subscripts and superscripts in Equation (13), for ease of notation, we used a MATLABmathsizesmall^®^-like notation for indexing matrices.

In other words, w acts as a feature (prototype) selector. Given this newly obtained dataset, it is possible to train a kernelised ν-SVM [64] whose multiple kernel has the form Equation (9) where each one has the form Equation (9), thus:

(14) $$K=\sum_{i=1}^{N_{R}}\beta_{i}\cdot \exp\left\{-\gamma_{i}\cdot {∥{\bar{D}}_{\mathrm{TR}}^{\left(i\right)}⊝{\bar{D}}_{\mathrm{TR}}^{\left(i\right)}∥}^{2}\right\},$$

where ⊝ denotes the pairwise difference. Hence, each dissimilarity representation is subject to a proper non-linear kernel ($N_{K}\equiv N_{R}$).

A genetic algorithm [65] acts as a wrapper method in order to automatically tune in a fully data-driven fashion the several free parameters introduced in this problem. The choice behind a genetic algorithm stems from them being widely famous in the context of derivative-free optimisation, embarrassingly easy to parallelise and for the sake of consistency with competing techniques (see Section 4.4). For our problem, the genetic code has the form:

(15) $$\left[\begin{array}{cccc} \nu & \beta & \gamma & w \end{array}\right],$$

where ν∈(0,1] is the SVM regularisation term, $\beta ={\left[\beta \right]}_{i=1}^{N_{R}}$ contains the kernel weights, $\gamma ={\left[\gamma_{i}\right]}_{i=1}^{N_{R}}$ contains the kernel shapes and w properly selects prototypes in the dissimilarity space, as described above.

For the sake of argument, it is worth remarking that there have been several attempts to use evolutionary strategies in order to tune multiple kernel machines: for example in [66] a genetic algorithm has been used in order to tune the kernel shapes (namely, γ), whereas in [67] both the kernel shapes and the kernel weights have been tuned by means of a (μ+λ) evolution strategy [68]. Conversely, the idea of using a genetic algorithm for prototypes selection in the dissimilarity space has been inherited from a previous work [44].

The fitness function to be maximised is the informedness *J* (also known as Youden’s index [69]) defined as:

(16) J=specificity+sensitivity−1,

which is, by definition, bounded in range [−1,1] (the closer to 1, the better). For the sake of comparison with other performance measures (e.g., accuracy, *F*-score and the like) which are, by definition, bounded in [0,1], the fitness function sees a scaled version of the informedness [23,24,25], hence:

(17) $$f_{1}\equiv \bar{J}=\frac{J-(-1)}{1-(-1)}=\frac{J+1}{2}\in \left[0,1\right].$$

The rationale behind using the informedness rather than other most common performance measures (mainly accuracy and *F*-score) is that the informedness is well suited for unbalanced classes without being biased towards the most frequent class (the same is not true for accuracy) and whilst considering also true negative predictions (the same is not true for *F*-score) [70].

By assuming that the full dissimilarity matrices are pre-evaluated beforehand, the objective function evaluation is performed for each individual from the current generation as follows:

1. The individual receives the $N_{R}$ full dissimilarity matrices between training data samples, i.e., $D_{\mathrm{TR}}^{\left(1\right)},\ldots ,D_{\mathrm{TR}}^{\left(N_{R}\right)}$ as in Equation (12);
2. According to the w portion of its genetic code (see Equation (15)), a subset of prototypes is selected, leading to the ‘reduced’ dissimilarity matrices between training data, i.e., ${\bar{D}}_{\mathrm{TR}}^{\left(1\right)},\ldots ,{\bar{D}}_{\mathrm{TR}}^{\left(N_{R}\right)}$ as in Equation (13);
3. Considering the β and γ values in its genetic code, the (multiple) kernel matrix is evaluated by using Equation (14);
4. A ν-SVM is trained using the regularisation term ν from the genetic code and the kernel matrix from step #3;
5. The individual receives the $N_{R}$ full dissimilarity matrices between training and validation data, each of which is computed by considering all possible 〈x,y〉-pairs where *x* belongs to the validation set and *y* belongs to the training set, i.e., $D_{\mathrm{VAL}}^{\left(1\right)},\ldots ,D_{\mathrm{VAL}}^{\left(N_{R}\right)}$ as in Equation (12);
6. The ‘reduced’ dissimilarity matrices are projected thanks to w, i.e., ${\bar{D}}_{\mathrm{VAL}}^{\left(1\right)},\ldots ,{\bar{D}}_{\mathrm{VAL}}^{\left(N_{R}\right)}$ as in Equation (13);
7. The (multiple) kernel matrix between training and validation data is evaluated thanks to β and γ, alike Equation (14);
8. The (multiple) kernel matrix from step #7 is fed to the SVM trained on step #4 and the predicted classes on the validation set are returned;
9. The fitness function is evaluated.

At the end of the evolution, the best individual (i.e., the one with best performances on the validation set) is retained and its final performances are evaluated on the test set.

Finally, it is worth remarking the rationale behind the proposed, structured, genetic code since a genetic code of the form Equation (15) allows, in a two-fold manner, a deeper a posteriori knowledge discovery phase. Indeed, using upfront good classification results (for the sake of reliability), by looking at β, it is possible to check which kernels (representations) are considered as the most important (higher weights) for the learning machine in order to solve the problem at hand. Similarly, by looking at w, it is possible to check which training set patterns have been selected as representatives and ask why those patterns have been selected instead of others, leading to a pattern-wise check (possibly with help by field-experts). Especially the latter a posteriori check might be troublesome if a huge number of representatives is selected. In order to alleviate this problem (if present), it is possible to re-state the fitness function (formerly (17)) by considering a convex linear combination between the performance index and the feature selector sparsity, hence:

(18) $$f_{2}=\omega \left(1-\bar{J}\right)+\left(1-\omega \right)\frac{\left|\{i:w_{i}=1\}\right|}{\left|w\right|},$$

where ω∈[0,1] in a user-defined parameter which tunes the convex linear combination by weighting the rightmost term (sparsity) against the leftmost term (performance). It is worth noting that whilst fitness (17) should be maximised, (18) should be minimised.

## 4. Tests and Results

### 4.1. Data Collection and Pre-Processing

The data retrieval processing can be summarised as follows. Using the Python BioServices library [71]:

1. The entire protein list for Escherichia coli str. K12 has been retrieved from UniProt [72];
2. This list has been cross-checked with Protein Data Bank [73] in order to discard unresolved proteins (i.e., proteins whose 3D structure is not available).

Then, using the BioPython library [74]:

1. .pdb files have been downloaded for all resolved proteins;
2. information such as the EC number and the measurement resolution (if present) have been parsed from the .pdb file header;
3. proteins having multiple EC numbers have been discarded.

Finally, using the BioPandas library [75]:

1. α-carbon atoms 3D coordinates have been parsed from each .pdb file;
2. In case of multiple equivalent models within the same .pdb file, only the first model is retained;
3. Similarly, for atoms having alternate coordinate locations, only the first location is retained.

**Figure 1:**
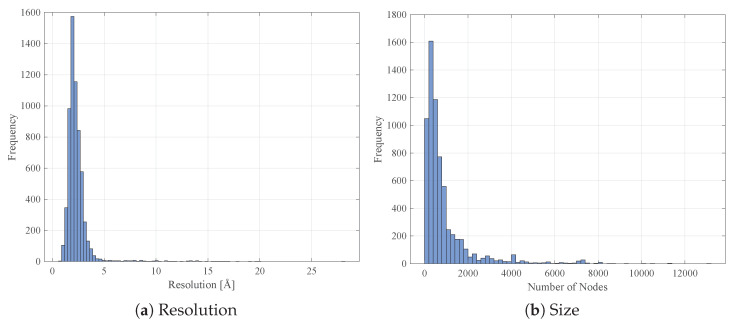
Distributions within the original 6685 proteins set.

After this retrieval stage, a total number of 6685 proteins has been successfully collected. Some statistics on the measurement resolutions and the number of nodes are sketched in Figure 1a,b, respectively.

In order to keep only good quality structures (with reliable atomic coordinates), all proteins with missing resolution in their respective .pdb files and proteins whose resolution is greater than 3Å have been discarded. Further, proteins having more than 1500 nodes have been discarded as well. These filtering procedures dropped the number of available proteins from 6685 to 4957. The class labels (EC number) distribution is summarised in Table 1.

For each of the 4957 available proteins, its respective eight representations (see Appendix A) have been evaluated using the following tools:

- The NetworkX library [76] (Python) for evaluating centrality measures ($X^{\left(2\right)}$) and the Vietoris–Rips complex ($X^{\left(1\right)}$);
- The Numpy and Scipy libraries [77,78] (Python) for several algebraic computations, mainly spectral decompositions for energy, Laplacian energy, heat trace, heat content invariants ($X^{\left(3\right)}$, $X^{\left(5\right)}$, $X^{\left(6\right)}$, $X^{\left(8\right)}$) and the homology group rank ($X^{\left(1\right)}$);
- The Rnetcarto (https://cran.r-project.org/package=rnetcarto) library (R) for network cartography ($X^{\left(4\right)}$).

As in previous works [45,46] the 7-class classification problem is cast into seven binary classification problems in one-against-all fashion, hence the *i*th classifier sees the *i*th class as positive and all other classes as negative. The eight representations $X^{\left(1\right)},\ldots ,X^{\left(8\right)}$ are split into training, validation and test set in a stratified manner in order to preserve labels’ distribution across splits. Thus, each of the seven classifiers sees a different training-validation-test split due to the one-against-all labels recoding. The genetic optimisation and classification stage has been performed in MATLABmathsizesmall^®^ R2018a using the built-in genetic algorithm and LibSVM [79] for ν-SVMs.

### 4.2. Computational Results with Fitness Function $f_{1}$

The first test suite sees $f_{1}$ (17) as the fitness function, hence the system aims at the maximisation of the (normalised) informedness.

The genetic algorithm has been configured to host 100 individuals for a maximum of 100 generations and each individual’s genetic code (upper/lower bounds and constraints, if any) is summarised in Table 2. At each generation, the elitism is set to the top 10% individuals; the crossover operates in a scattered fashion; the selection operator follows the roulette wheel heuristic and the mutation adds to each real-valued gene (ν,β,γ) a random number extracted from a zero-mean Gaussian distribution whose variance shrinks as generations go by, whereas it acts in a flip-the-bit fashion for boolean-valued genes (w).

Table 3 shows the performances obtained by the proposed Multiple Kernels over Multiple Dissimilarities (MKMD, for short) approach using the fitness function $f_{1}$. Due to randomness in genetic optimisation, five runs have been performed for each classifier and the average results are shown. Figures of merit include:

- $\mathrm{Accuracy}=\frac{TP+TN}{TP+FP+TN+FN}$;
- $\mathrm{Precision}=\frac{TP}{TP+FP}$;
- $\mathrm{Recall}\;\left(\mathrm{Sensitivity}\right)=\frac{TP}{TP+FN}$;
- (Normalised)Informedness as in Equation (17);
- AreaUndertheCurve (AUC), namely the area under the Receiver Operating Characteristic (ROC) curve [80];

where TP, TN, FP and FN indicate true positives, true negatives, false positives and false negatives, respectively.

**Figure 2:**
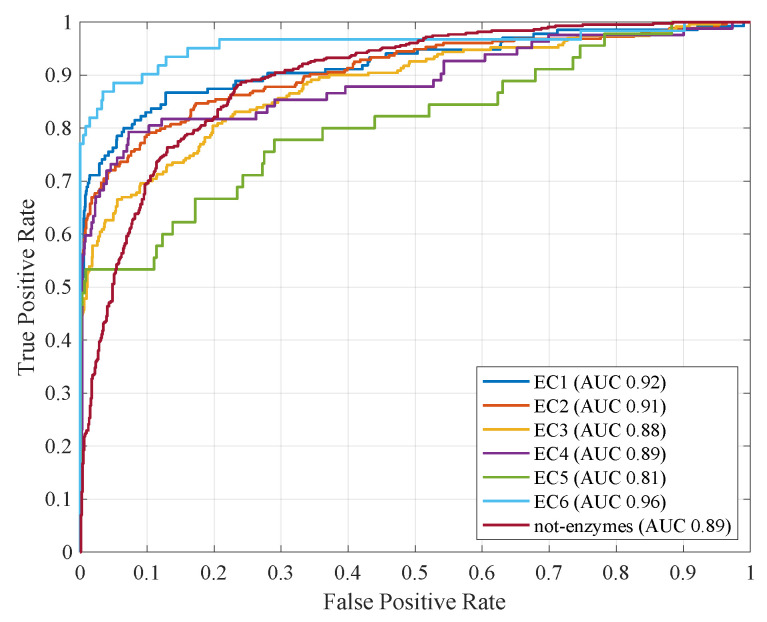
ROC curves with fitness function $f_{1}$. In brackets, the respective AUC values.

Similarly, Figure 2 shows the ROC curves for all classifiers by considering their respective run with greatest AUC.

### 4.3. Computational Results with Fitness Function $f_{2}$

**Figure 3:**
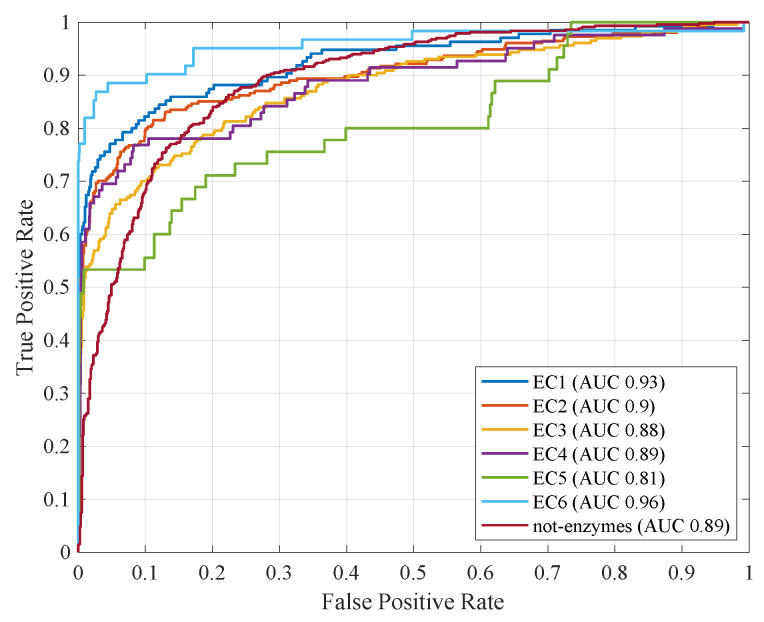
ROC curves with fitness function $f_{2}$ and ω=0.5. In brackets, the respective AUC values.

These experiments see the fitness function $f_{2}$ (Equation (18)) in lieu of $f_{1}$ (Equation (17)), where the weighting parameter ω is set to 0.5 in order to give the same importance to performances and sparsity. In order to ensure a fair comparison with the previous analysis, the same training-validation-test splits have been used for all seven classifiers, along with the same genetic algorithm setup (genetic code, number of individuals and generations, genetic operators). Table 4 shows the average performances obtained by the seven classifiers across five genetic algorithm runs. As in the previous case, Figure 3 shows the ROC curves for all classifiers by considering their respective run with greatest AUC.

### 4.4. Benchmarking against a Clustering-Based One-Class Classifier

In order to properly benchmark the proposed MKMD system, a One-Class Classification System (hereinafter OCC or OCC_System) capable of exploiting multiple dissimilarities is used. This classification system has been initially proposed in [81] and later used for modelling complex systems such as smart grids [81,82,83] and protein networks [44].

The main idea in order to build a model through the One-Class Classifier is to use a clustering-evolutionary hybrid technique [81,82]. The main assumption is that similar protein types have similar chances of generating a specific class, reflecting the cluster model. Therefore, the core of the recognition system is a custom-based dissimilarity measure computed as a weighted Euclidean distance, that is:

(19) $$d\left({\overset{ˇ}{\vec{x}}}_{1},{\overset{ˇ}{\vec{x}}}_{2};\vec{W}\right)=\sqrt{{\left({\overset{ˇ}{\vec{x}}}_{1}⊖{\overset{ˇ}{\vec{x}}}_{2}\right)}^{T}{\vec{W}}^{T}\vec{W}\left({\overset{ˇ}{\vec{x}}}_{1}⊖{\overset{ˇ}{\vec{x}}}_{2}\right)},$$

where ${\overset{ˇ}{\vec{x}}}_{1},{\overset{ˇ}{\vec{x}}}_{2}$ are two generic patterns and $\vec{W}$ is a diagonal matrix whose elements are generated through a suitable vector of weights $\vec{w}$. The dissimilarity measure is component-wise, therefore the ⊖ symbol represents a generic dissimilarity measure, tailored on each pattern subspace, that has to be specified depending on the semantic of data at hand.

In this study, patterns are represented by dissimilarity vectors extracted from each sub-dissimilarity matrix, one for each feature adopted to describe the protein (see Section 2). In other words, patterns pertain to a suitable dissimilarity space.

The decision region of each cluster $C_{i}$ is constructed around the medoid $c_{i}$ bounded by the average radius $\delta (C_{i})$ plus a threshold σ, considered together with the dissimilarity weights $\vec{w}=diag\left(\vec{W}\right)$ as free parameters. Given a test pattern $\overset{ˇ}{\vec{x}}$ the decision rule consists in evaluating whether it falls inside or outside the overall target decision region, by checking whether it falls inside the closest cluster. The learning procedure consists in clustering the training set $D_{\mathrm{TR}}$ composed by target patterns, adopting a standard genetic algorithm in charge of evolving a family of cluster-based classifiers considering the weights $\vec{w}$ and the thresholds of the decision regions as search space, guided by a proper objective function. The latter is evaluated on the validation set $D_{\mathrm{VAL}}$, taking into account a linear combination of the accuracy of the classification (that we seek to maximise) and the extension of the thresholds (that should be minimised). Note that in building the classification model we use only target patterns, while non-target ones are used in the cross-validation phase, hence the adopted learning paradigm is the One-Class classification one [84,85]. Moreover, in order to outperform the well-known limitations of the initialization of the standard *k*-means algorithm, the OCC_System initializes more than one instance of the clustering algorithm with random starting representatives, namely medoids, since the OCC_System is capable of dealing with arbitrarily structured data [86,87,88]. At test stage (or during validation) a voting procedure for each cluster model is performed. This technique allows building a more robust proteins model.

**Figure 4:**
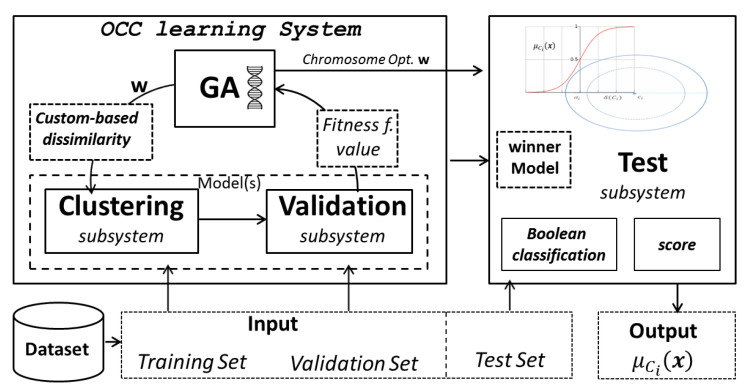
Schematic of the classification system able to learn a classification model for each positive class. The model provides the crisp decision as well as a score (a real number) encoding the decision reliability.

Figure 4 shows the schematic representing the core subsystems of the proposed OCC_System, such as the ones performing the clustering procedure and the genetic algorithm. Moreover, it is shown the Test subsystem, where given a generic test pattern and given a learned model, it is possible to associate a score value (soft-decision) besides the Boolean decision. Hence, we equip each cluster $C_{i}$ with a suitable membership function, denoted in the following as $\mu_{C_{i}}\left(\cdot \right)$. In practice, we generate a fuzzy set [89] over $C_{i}$. The membership function allows quantifying the uncertainty (expressed by the membership degree in [0,1]) of a decision about the recognition of a test pattern. Membership values close to either 0 or 1 denote “certain” and hence reliable decisions. When the membership degree assigned to a test pattern is close to 0.5, there is no clear distinction about the fact that such a test pattern is really a target pattern or not (regardless of the correctness of the Boolean decision).

For this purpose, we adopt a parametric sigmoid model for $\mu_{C_{i}}\left(\cdot \right)$, which is defined as follows:

(20) $$\mu_{C_{i}}\left(x\right)=\frac{1}{1+\exp\{\left(d\left(c_{i},x\right)-b_{i}\right)/a_{i}\}},$$

where $a_{i},b_{i}\geq 0$ are two parameters specific to $C_{i}$, and d(·,·) is the dissimilarity measure (19). Notably, $a_{i}$ is used to control the steepness of the sigmoid (the lower the value, the faster the rate of change), and $b_{i}$ is used to translate the function in the input domain. If a cluster (that models a typical protein found in the training set) is very compact, then it describes a very specific scenario. Therefore, no significant variations should be accepted to consider test patterns as members of this cluster. Similarly, if a cluster is characterised by a wide extent, then we might be more tolerant in the evaluation of the membership. Accordingly, the parameter $a_{i}$ is set equal to $\delta (C_{i})$. On the other hand, we define $b_{i}=\delta \left(C_{i}\right)+\sigma_{i}/2$. This allows us to position the part of the sigmoid that changes faster right in-between the area of the decision region determined by the dissimilarity values falling in $\left[B\left(C_{i}\right)-\sigma_{i},B\left(C_{i}\right)\right]$, where in turn $B\left(C_{i}\right)=\delta \left(C_{i}\right)+\sigma_{i}$ is the boundary of the decision region related to the *i*th cluster.

Finally, the soft decision function, s(·), is defined as

(21) $$s\left(\bar{x}\right)=\mu_{C^{*}}\left(\bar{x}\right),$$

where $C^{*}$ is the cluster where the test (target) pattern falls.

With the aim of making a synthesis, we remark that the OCC_System works in two phases:

1. Learning a cluster model of proteins through a suitable dataset divided into two disjoint sets, namely training and validation set;
2. Using the learned model in order to recognise or classify unseen proteins drawn from the test set, assigning to each pattern a probability value.

The OCC parameters defining the model are optimised by means of a genetic algorithm guided by a suitable objective function that takes into account the classification accuracy. For the sake of comparison, the same genetic operators (selection, mutation, crossover, elitism) as per the MKMD system and have been considered (see Section 4.2). As concerns the complexity of the model, measured as the cardinality of the partition *k*, we choose a suitable value k=120.

**Figure 5:**
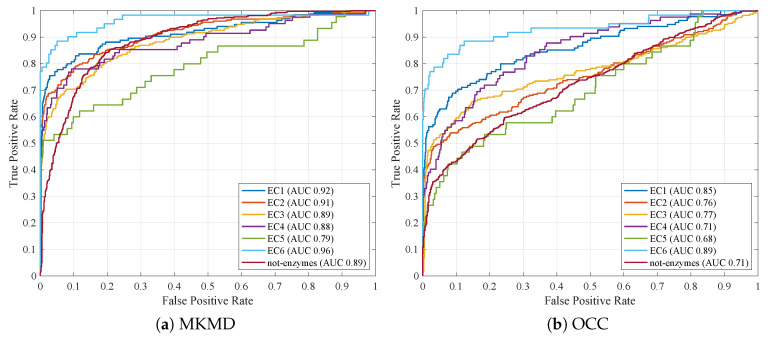
ROC curves comparison (best run for all classes). In brackets, the respective AUC values.

Table 5 shows the comparison between the OCC_System and the MKMD approach. In order to ensure a fair comparison, since the OCC_System does not perform representatives selection in the dissimilarity space, in the MKMD genetic code (cf. Equation (15)), the weights vector w has been removed and all weights have been considered unitary (i.e., no representative selection). Similarly, Figure 5b and Figure 5a show the ROC curves for OCC and MKMD, respectively.

From Table 5 is evident that MKML outperforms OCC in terms of accuracy, informedness and AUC (see also the ROC curves in Figure 5b and Figure 5a), but a clear winner does not exist as regards precision and recall. As regards the structural complexity, OCC is bounded by the number of clusters *k*, whereas MKMD is bounded by the number of support vectors as returned by the training phase [24]. Indeed, the computational burden required to classify new test data is given by:

- The pairwise distances between the test data and the *k* clusters centres (for OCC);
- The dot product between the test data and the support vectors (for MKMD).

Specifically, for OCC, a suitable number of 120 clusters has been defined for all classes, whereas the training phase for MKMD returned an average of 1300 support vectors (∼52% of the training data) for class 1, 1881 support vectors (∼76%) for class 2, 1745 support vectors (∼70%) for class 3, 1213 support vectors (∼49%) for class 4, 767 support vectors (∼31%) for class 5, 864 support vectors (∼35%) for class 6 and 1945 support vectors (∼78%) for class 7. In conclusion, whilst MKMD outperforms OCC in terms of performances, the latter outperforms the former in terms of structural complexity.

### 4.5. Comparing against Previous Works

In Table 6 are reported the performances (in terms of AUC only, for the sake of shorthand) between the proposed MKMD approach with fitness function $f_{1}$ (Table 3), with fitness function $f_{2}$ (Table 4) and with no representatives selection in the embedding space (Table 5) against our previous studies for solving the same classification problem. For the sake of completeness, the results obtained by OCC (Table 5) are also included.

In [44], two experiments have been performed: the first relied on the Dissimilarity Matrix Embedding (DME) by considering different protein representations (similar to the ones considered in this work) and the second one relied on OCC being able to explore those different representations simultaneously (alike this work). There are three main differences between this work and [44]: first, the set of representations is different; second, we only managed to solve the binary classification problem between enzymes and not-enzymes; third, the set of considered proteins is different. In fact, in [44], we performed an additional filtering stage in order to select (for the same UniProt ID) only the PDB entry with best resolution: we found that this heavily limits the number of protein samples available, possibly reducing the learning capabilities.

In [45,46] we used the sampled spectral density of the protein contact networks (more information can be found in Appendix A.8) and the Betti numbers (more information can be found in Appendix A.1), respectively: the results in Table 6 feature the same proteins set used in this work. Indeed, thanks to the observation above, experiments have been repeated with an augmented number of protein samples [90,91].

Results in Table 6 highlight that:

1. Avoiding to filter out PDB structures by considering only the best resolution for a given UniProt ID (as carried out also in this work) helps in improving classification models: indeed, performances from [44] are amongst the lowest ones;
2. The proposed MKMD approach, regardless of the fitness function and/or representative selection, outperforms all competitors for all EC classes (including not-enzymes).

### 4.6. On the Knowledge Discovery Phase

Apart from the good generalisation capabilities, it is worth remarking that an interesting aspect of the proposed multiple kernel approach is the two-fold knowledge discovery phase:

1. By analysing the kernel weights β, it is possible to determine the most important representations for the problem at hand;
2. By analysing w, namely the binary vector in charge of selecting prototypes from the dissimilarity space, it is possible to determine and analyse the patterns (proteins, in this case) elected as prototypes.

Let us start our discussion from the latter point. From a chemical viewpoint, proteins are linear hetero-polymers in the form of non-periodic sequences of 20 different monomers (amino-acids residues). While artificial polymers (periodic) are very large extended molecules forming a matrix, the majority of proteins fold as self-contained water-soluble structures. Thus, we can consider the particular linear arrangement of amino-acid residues as a sort of ‘recipe’ for making a water-soluble polymer with a well-defined three-dimensional architecture [92]. “Well-defined three-dimensional structure” should not be intended as a ‘fixed architecture’: many proteins appear as partially or even totally disordered when analysed with spectroscopic methods. This apparent disorder corresponds to an efficient organisation as for protein physiological role giving to the molecule the possibility to adapt to rapidly changing microenvironment conditions [93].

This implies the two main drivers of amino-acid residues 3D arrangement (from where the particular properties of relative contact networks derive) are:

1. To efficiently accomplish the task of being water soluble while maintaining a stable structure (or dynamics);
2. To allow for an efficient spreading of the signal across amino-acid residues contact network so to sense relevant microenvironment changes and to reshape accordingly—allosteric effect, see [94].

Currently, we have only a coarse-grain knowledge of such complex tasks, and biochemists are still very far to be able to reproduce this behaviour by synthetic constructs.

The ability to catalyse a specific class of chemical reactions (the property the EC classification is based upon), while being crucial for the biological role of protein molecules is, from the point of view of topological and geometrical proteins structure, only a very minor modulation of their global shape [92]. Notwithstanding that, the thorough analysis of representative proteins (thus pivotal for discrimination) can give us some general hints, not only confined to the specific classification task, but extending to all the ’hard’ classification problems based upon very tiny details of the statistical units.

**Figure 6:**
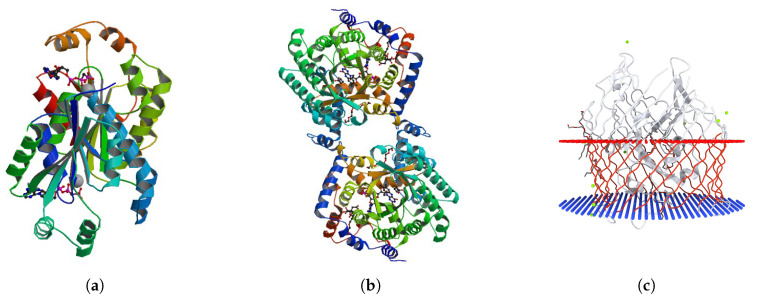
Three basic patterns in protein 3D structures. (**a**) Transferase—PDB ID 1KOF, (**b**) Proline dehydrogenase (oxidoreductase)—PDB ID 3E2R, (**c**) Transport Protein (Non-Enzyme)—PDB ID 3RGM.

Looking at the representative proteins (hence, endowed with meaningful discriminative power) in Table A1, Table A2, Table A3, Table A4, Table A5, Table A6 and Table A7 (Appendix B) we immediately note that the pivotal proteins come from all the analysed EC categories and not only from the specific class to be discriminated. This is expected by the absence of a simple form-function relation, hence they can be considered as an ‘emergent property’ of the discrimination task. The presence of molecules of different classes crucial for a specific category modelling and thus the image in light of a peculiar strategy adopted by the system is analogue to the use of ‘paired samples’ in statistical investigation [95,96]. When in presence of only minor details discriminating statistical units pertaining to different categories, the only possibility to discriminate is to adopt a paired samples strategy in which elements of a category is paired with a very similar example of another category so to rely on their differences (on a sample-by-sample basis) instead of looking for a general ‘class-specific’ properties. This is the case of proteins whose general shape is only partially determined by the chemical reaction they catalyse: looking at the 3D structures of relevant proteins, we can easily verify they pertain to three basic patterns (Figure 6):

1. Cyclic pattern with an approximately spherical symmetry (Figure 6a);
2. A globular pattern with ‘duplication’: protein can be considered as two identical half-structures (Figure 6b);
3. Elongated non-cyclic pattern, typical of membrane-bound proteins (Figure 6c).

Even if the three above-mentioned patterns have slightly different relative frequencies in the EC classes (e.g., pattern 3 is more frequent in non-enzymatic proteins), they are present in all the analysed classes so allowing for the ‘between-categories’ sample-by-sample pairing mentioned above.

This peculiar situation is in line with current biochemical knowledge (minimal effect exerted by catalysed reaction on global structure) and it is a relevant proof-of-concept of both the reliability of the classification solution and of the power of the proposed approach. On the other hand, it is very hard to de-convolve the discriminating structural nuances from the obtained solution that, as it is, only confirms the presence of ‘tiny and still unknown’ structural details linked to the catalytic activity of the studied molecules.

**Figure 7:**
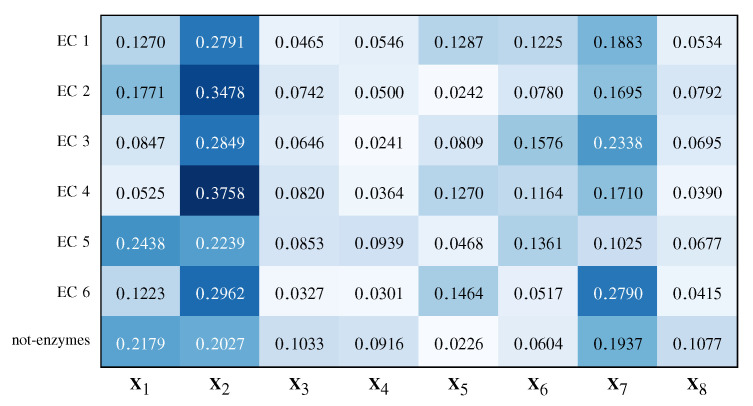
Average kernel weights vectors β.

As regards the former point, Figure 7 shows the average weights vector β across the aforementioned five runs for ω=0.5, showing that the MKMD approach considers for almost all classes centrality measures ($X_{2}$) and the protein size ($X_{7}$) as the most relevant representations, followed by the Betti numbers sequence ($X_{1}$), heat content invariants ($X_{5}$) and heat kernel trace ($X_{6}$).

It is worth noting that enzymes have a more pronounced allosteric effect with respect to non-enzymatic structures. This is a consequence of the need to modulate chemical kinetics according to microenvironment conditions—allostery is the modulating effect of a modification happening in a site different from catalytic site on the efficiency of the reaction [97]. Allostery implies an efficient transport of the signal along protein structure and it was discovered to be efficiently interpreted in terms of PCN descriptors [98] thus, the observed kernel weights fit well with the current biochemical knowledge.

## 5. Conclusions

In this paper, we proposed a classification system able to explore simultaneously multiple representations following an hybridisation between multiple kernel learning and dissimilarity spaces, hence exploiting the discriminative power of kernel methods and the customisability of dissimilarity spaces.

Specifically, several representations are treated using their respective dissimilarity representations and combined in a multiple kernel fashion, where each kernel function considers a specific dissimilarity representation. A genetic algorithm (although any derivative-free evolutive metaheuristic can be placed instead) is able to simultaneously select suitable representatives in the dissimilarity space and tune the kernel weights, allowing a two-fold a posteriori knowledge discovery phase regarding the most suitable representations (higher kernel weights) and the patterns elected as prototypes in the dissimilarity space.

The proposed MKMD system has been applied for solving a real-world problem, namely protein function prediction, with satisfactory results, greatly outperforming our previous works in which graph-based descriptors extracted from PCNs have been tested for solving the very same problem. Further, the proposed system has been benchmarked against a One-Class Classifier, also able to simultaneously explore multiple dissimilarities: whilst the former outperforms the latter in terms of accuracy, AUC and informedness, a clear winner between the two methods does not exist in terms of precision and recall.

As far as the two-fold knowledge discovery phase for the proposed application is concerned, results both in terms of selected representatives in the dissimilarity space and weights automatically assigned to different representations are in line with current biological knowledge, showing the reliability of the proposed system.

Furthermore, due to its flexibility, the proposed system can be applied to any input domain (not necessarily graphs), provided that several representations can be extracted by the structured data at hand and that suitable dissimilarity measures can be defined for such heterogeneous representations.

## Figures and Tables

**Table 1:** Classes distribution within the filtered 4957 proteins set.

								Total
**Class**	EC1	EC2	EC3	EC4	EC5	EC6	not-enzymes	
**Count**	540	1017	919	329	182	244	1726	4957
**Percentage**	10.89	20.52	18.54	6.64	3.67	4.92	34.82	100%

**Table 2:** Genetic algorithm parameters description.

Parameter	Bounds	Contraints
ν	(0,1] by definition	
β	$\beta_{i}\in \left[0,1\right],\;\forall i=1,\ldots ,N_{R}$	$\sum_{i=1}^{N_{R}}\beta_{i}=0$
γ	$\gamma \in \left(0,100\right],\;\forall i=1,\ldots ,N_{R}$	
w	$w_{i}\in \left\{0,1\right\},\;\forall i=1,\ldots ,\left|D_{\mathrm{TR}}\right|$	

**Table 3:** Test Set Performances with Fitness Function $f_{1}$.

Class	Performances					Complexity
	Accuracy	Precision	Recall	Informedness ${}^{†}$	AUC	Sparsity
1 (EC1)	0.95	0.87	0.68	0.83	0.92	49.43
2 (EC2)	0.91	0.88	0.66	0.82	0.90	49.62
3 (EC3)	0.90	0.84	0.58	0.78	0.88	49.48
4 (EC4)	0.97	0.90	0.56	0.78	0.88	49.42
5 (EC5)	0.98	0.83	0.44	0.72	0.78	50.78
6 (EC6)	0.99	0.94	0.76	0.88	0.95	49.28
7 (not-enzymes)	0.82	0.77	0.70	0.79	0.89	50.52

${}^{†}$ Normalised.

**Table 4:** Test set performances with fitness function $f_{2}$ and ω=0.5.

Class	Performances					Complexity
	Accuracy	Precision	Recall	Informedness ${}^{†}$	AUC	Sparsity
1 (EC1)	0.95	0.86	0.69	0.84	0.92	33.08
2 (EC2)	0.91	0.88	0.67	0.82	0.90	32.48
3 (EC3)	0.90	0.83	0.57	0.77	0.87	29.94
4 (EC4)	0.97	0.88	0.54	0.77	0.88	33.89
5 (EC5)	0.98	0.85	0.45	0.73	0.79	35.54
6 (EC6)	0.98	0.91	0.76	0.88	0.95	35.38
7 (not-enzymes)	0.82	0.77	0.69	0.79	0.88	33.37

${}^{†}$ Normalised.

**Table 5:** Test set performances with the one-class classifier.

Class	Classifier	Performances				
		Accuracy	Precision	Recall	Informedness ${}^{†}$	AUC
1 (EC1)	OCC	0.92	**0.97**	0.35	0.67	0.85
	MKMD	**0.95**	0.88	**0.67**	**0.83**	**0.91**
2 (EC2)	OCC	0.83	0.87	0.45	0.69	0.76
	MKMD	**0.91**	**0.89**	**0.66**	**0.82**	**0.91**
3 (EC3)	OCC	0.83	**0.86**	0.49	0.70	0.77
	MKMD	**0.90**	0.84	**0.57**	**0.77**	**0.88**
4 (EC4)	OCC	0.68	0.60	**0.78**	0.61	0.72
	MKMD	**0.97**	**0.89**	0.53	**0.76**	**0.87**
5 (EC5)	OCC	0.85	0.75	0.37	0.62	0.69
	MKMD	**0.98**	**0.82**	**0.44**	**0.72**	**0.78**
6 (EC6)	OCC	0.97	0.96	0.57	0.78	0.88
	MKMD	**0.99**	**0.92**	**0.77**	**0.88**	**0.95**
7 (not-enzymes)	OCC	0.68	0.60	**0.78**	0.61	0.72
	MKMD	**0.82**	**0.78**	0.68	**0.79**	**0.88**

${}^{†}$ Normalised.

**Table 6:** Comparison (in terms of AUC) between the proposed MKMD approach and previous studies.

Approach	EC1	EC2	EC3	EC4	EC5	EC6	Not-Enzymes
DME + Logistic Regression [44]	–	–	–	–	–	–	0.62
DME + SVM [44]	–	–	–	–	–	–	0.64
DME + Naïve Bayes [44]	–	–	–	–	–	–	0.62
DME + Decision Tree [44]	–	–	–	–	–	–	0.60
DME + Neural Network [44]	–	–	–	–	–	–	0.63
OCC [44]	–	–	–	–	–	–	0.63
Feature Generation via Betti Numbers + SVM [46]	0.79	0.75	0.73	0.73	0.46	0.77	0.77
Feature Generation via Spectral Density + SVM [45]	0.85	0.82	0.85	0.81	0.59	0.81	0.82
MKMD with $f_{1}$ (Table 3)	0.92	0.90	0.88	0.88	0.78	0.95	0.89
MKMD with $f_{2}$ (Table 4)	0.92	0.90	0.87	0.88	0.79	0.95	0.88
MKMD with no representative selection (Table 5)	0.91	0.91	0.88	0.87	0.78	0.95	0.88
OCC (Table 5)	0.85	0.76	0.77	0.72	0.69	0.88	0.72

## Abbreviations

The following abbreviations are used in this manuscript:

AUC	Area Under the Curve
DME	Dissimilarity Matrix Embedding
MKMD	Multiple Kernels over Multiple Dissimilarities
OCC	One-Class Classification (also OCC_System)
PCN	Protein Contact Networks
PDB	Protein Data Bank
ROC	Receiver Operating Characteristic
SVM	Support Vector Machine

## Appendix A. Selected Representations

The set of eight representations $X^{\left(1\right)},\ldots ,X^{\left(8\right)}$ used to characterise PCNs are described in the following eight subsections.

### Appendix A.1. Betti Numbers

Topological Data Analysis [99,100] is a novel data analysis approach useful whenever data can be described by topological structures (networks) as it consists in a set of techniques in order to extract information from data (starting from topological information) by means of dimensionality reduction, manifold estimation and persistent homology in order to study how components lying in a multi-dimensional space are connected (e.g., in terms of loops and multi-dimensional surfaces). One can start either from so-called point clouds, where objects are described by their coordinates in a multi-dimensional space equipped with notion of distance, or by explicitly providing the pairwise distance matrix between objects. Hereinafter, the former case is considered.

The most intuitive scenario in order to study how components lying in a multi-dimensional space are connected is (trivially) by studying the connectivity itself. To this end, it is worth defining simplices as (multi-dimensional) topological objects which can be extracted from a given topological space X: points, lines, triangles and tetrahedrons are (for example) 0-dimensional, 1-dimensional, 2-dimensional, 3-dimensional simplices and, obviously, higher-order analogues exist. Simplices can be seen as descriptors of the space under analysis, thus worthy of attention when studying X. Starting from simplices, it is possible to define simplicial complexes as properly-constructed collection of simplices able to capture the multi-scale organisation (or multi-way relations) in complex networks [101,102,103]. The two seminal examples of simplicial complexes are the Čech complex and the Vietoris–Rips complex [99,100,104,105], however due to its intuitiveness and lighter computational complexity, one in practice uses the latter. The Vietoris–Rips complex can be built according to the following rule: initially, all 0-dimensional simplices belong to the complex, then a given set of *k* points forms a (k−1)-dimensional simplicial complex to be included in the Vietoris–Rips complex if the pairwise distances are all less than or equal to a user-defined threshold ϵ.

The homology of a simplicial complex can be described by its Betti numbers. Formally, the *i*th Betti number is the rank of the *i*th homology group in the simplicial complex. Informally, the *i*th Betti number corresponds to the number of *i*-dimensional ‘holes’ in a topological surface. In this work, 3-dimensional graphs are considered and the first three Betti numbers have the following interpretations: the 0th Betti number is the number of connected components, the 1st Betti number is the number of 1-dimensional (circular) holes, the 2nd Betti number is the number of 2-dimensional holes (cavities). The Betti numbers vanish after the spatial dimension.

From the above Vietoris–Rips complex definition, it is clear that the choice of ϵ is critical as it somewhat defines the resolution of the simplicial complex. In many cases, one builds a sequence of Vietoris–Rips complexes as ϵ varies in order to study how ‘holes’ appear and disappear as the resolution changes and then selects a desired value $\epsilon^{⋆}$ by studying the ‘holes’ lifetime in order to obtain a useful homology summary: in algebraic topology, this concept is known as persistence [106].

Instead of having a ‘topological summary’, following a previous work [46], the rationale is to keep proper track of the number of holes as ϵ changes. To this end, the range ϵ∈[4,8] with sampling step 1 is considered, according to the PCN connectivity range. Hence, the first representation $X^{\left(1\right)}$ sees each protein as a 15-length integer-valued vector obtained by the concatenation of $b_{4},b_{5},b_{6},b_{7},b_{8}$, where $b_{i}$ is (in turn) a 3-dimensional vector containing the first three Betti numbers for ϵ=i.

Technically speaking, for a given ϵ, the Vietoris–Rips complex can be evaluated in two steps [107]:

1. Build the Vietoris–Rips neighbourhood graph $G_{VR}\left(V,E\right)$: an undirected graph where edges between two nodes, say $v_{i},v_{j}\in V$, are scored if $d(v_{i},v_{j})\leq \epsilon$;
2. The set of maximal cliques in $G_{VR}$ form the Vietoris–Rips complex.

Let $\partial_{k}:S_{k}\to S_{k-1}$ be the boundary operator, an incidence-like matrix which maps $S_{k}$ (i.e., the set of simplices of order *k*) with the set of simplices of order k−1. The *k*-order homology group is defined as [108]:

(A1) $$H_{k}=\ker\left\{\partial_{k}\right\}/\mathrm{im}\left\{\partial_{k+1}\right\},$$

where ker{·} and im{·} denote the kernel and image operators. The rank of $H_{k}$, namely the *k*^th^ Betti number is then defined as [102]:

(A2) $$b^{\left(k\right)}=\mathrm{rank}\left\{\ker\left\{\partial_{k}\right\}\right\}-\mathrm{rank}\left\{\mathrm{im}\left\{\partial_{k+1}\right\}\right\}$$

or, thanks to the Rank–Nullity theorem [109]:

(A3) $$b^{\left(k\right)}=\left(\dim\left\{\partial_{k}\right\}-\mathrm{rank}\left\{\mathrm{im}\left\{\partial_{k}\right\}\right\}\right)-\mathrm{rank}\left\{\mathrm{im}\left\{\partial_{k+1}\right\}\right\},$$

where the rank of the image corresponds to the plain matrix rank in linear algebra.

### Appendix A.2. Centrality Measures

In graph theory and network analysis, centrality measures indicate the node/edge importance with respect to a given criterion. Let G=(V,E) be a graph and let V and E be the set of nodes and edges, respectively. The following centrality measures are considered:

- The degree centrality [110] $DC(v_{i})$ for node $v_{i}\in V$, defined as the percentage of nodes connected to it:
  (A4) $$DC\left(i\right)=\frac{1}{|V|-1}\sum_{j}A_{i,j},$$
  where A is the adjacency matrix, defined as in Equation (A22). The normalisation coefficient $\frac{1}{|V|-1}$ takes into account the maximum attainable degree in a simple graph, thus making the degree centrality in Equation (A4) independent from the number of nodes in the graph;
- The eigenvector centrality [110] highly rank nodes whether they are connected to other high-rank nodes. Formally, the eigenvector centrality $e_{i}$ for node $v_{i}\in V$ is given by:
  (A5) $$e_{i}=\frac{1}{\lambda }\sum_{j}A_{j,i}e_{j}\;,$$
  where λ≠0 is a scalar constant. Equation (A5) can be re-written in matrix form as:
  (A6) λe=eA.
  Hence, the eigenvector centrality vector e is the left-hand eigenvector of the adjacency matrix A associated with the eigenvalue λ. According to the Perron–Frobenius theorem, by choosing λ as the largest (in absolute value) eigenvalue of A, the solution e is unique and all its entries are positive;
- The PageRank centrality [110] $p_{i}$ for node $v_{i}\in V$ is given by:
  (A7) $$p_{i}=\alpha \sum_{j}\frac{A_{j,i}}{D(v_{j})}p_{j}+\frac{1-\alpha }{\left|V\right|},$$
  where α is a scalar constant (usually α=0.85) and $D(v_{j})$ is the degree of node $v_{j}$. It is worth remarking the difference between degree and degree centrality: the degree is the number of nodes connected to a given node (namely Equation (A4) without the normalisation term), whereas the degree centrality includes the normalisation term. As in the eigenvector centrality case, Equation (A7) can be re-written in matrix form as:
  (A8) $$p=\alpha pD^{-1}A+\beta ,$$
  where $D^{-1}$ is a diagonal matrix whose *i*th element equals $1/D(v_{i})$ and β is a vector whose elements are all equal to $\frac{1-\alpha }{\left|V\right|}$;
- The Katz centrality [110,111] $k_{i}$ for node $v_{i}\in V$ is given by:
  (A9) $$k_{i}=\alpha \sum_{j}A_{i,j}k_{j}+\beta ,$$
  where β controls the initial centrality (first neighbourhood weights) and $\alpha <1/\lambda_{\max}$ attenuates the importance with respect to higher-order neighbours (in turn, $\lambda_{\max}$ is the largest eigenvalue of A). It is worth noting that if $\alpha =1/\lambda_{\max}$ and β=0, the Katz centrality equals the eigenvector centrality;
- The closeness centrality [110] $CC(v_{i})$ for node $v_{i}\in V$ is the inverse sum of shortest path distances between node $v_{i}\in V$ and all other n−1 reachable nodes. Formally:
  (A10) $$CC\left(v_{i}\right)=\frac{n-1}{|V|-1}\frac{n-1}{\sum_{j=1}^{n-1}\delta \left(v_{i},v_{j}\right)},$$
  where δ(·,·) indicates the shortest path distance. The normalisation factor takes into account the graph size in order to allow comparison between nodes of graphs having different sizes, also in case of multiple connected components [112]. Indeed, *n* can be seen as the number of nodes in the connected component in which $v_{i}$ lies. In case of one connected component, the scale factor (n−1)/(|V|−1) can be neglected since n=|V|;
- The betweenness centrality [110] $BC(v_{i})$ quantifies how many times a given node $v_{i}\in V$ acts as a bridge along the shortest paths between any two nodes:
  (A11) $$BC\left(v_{i}\right)=\sum_{v_{i}\neq v_{j}\neq v_{k}}\frac{s^{\left(v_{i}\right)}\left(v_{j},v_{k}\right)}{s(v_{j},v_{k})},$$
  where $s(v_{j},v_{k})$ is the number of shortest paths from $v_{i}$ to $v_{j}$ and $s^{\left(v_{i}\right)}\left(v_{j},v_{k}\right)$ is the number of shortest paths from $v_{i}$ to $v_{j}$ passing through $v_{i}$. As in the closeness centrality case, it is often customary to normalise the betweenness centrality in order to avoid dependency from the number of nodes, thus:
  (A12) $$BC\left(v_{i}\right):=\frac{2\cdot BC(v_{i})}{\left(|V|-1)\cdot (|V|-2\right)};$$
- The edge betweenness centrality [113] $EBC(e_{i})$ is the edge counterpart of the “standard” (node) betweenness centrality as it quantifies how many times a given edge $e_{i}\in E$ acts as a bridge along the shortest paths between two nodes:
  (A13) $$EBC\left(e_{i}\right)=\sum_{v_{i},v_{j}\in V}\frac{s^{\left(e_{i}\right)}\left(v_{i},v_{j}\right)}{s(v_{i},v_{j})},$$
  where $s^{\left(e_{i}\right)}\left(v_{i},v_{j}\right)$ is the number of shortest paths between nodes $v_{i}$ and $v_{j}$ passing through edge $e_{i}$ and $s(v_{i},v_{j})$ is the total number of shortest paths between nodes $v_{i}$ and $v_{j}$. As in the “standard” betweenness centrality, the edge betweenness centrality can be normalised as follows:
  (A14) $$EBC\left(e_{i}\right):=\frac{2\cdot EBC(e_{i})}{\left(|V|-1)\cdot |V\right|};$$
- The load centrality [113,114] $LC(v_{i})$ for node $v_{i}\in V$ is the percentage of the total number of shortest paths passing through $v_{i}$;
- The edge load centrality $ELC(e_{i})$ for edge $e_{i}\in E$ is the edge-related counterpart of the load centrality (like betweenness vs. edge betweenness): it is defined as the percentage of the total number of shortest paths crossing edge $e_{i}$;
- The subgraph centrality [115] $SC(v_{i})$ for node $v_{i}\in V$ is the sum of (weighted) closed walks (i.e., connected subgraphs) starting and ending at $v_{i}$ (the longer the walk, the lower the weight). It can be evaluated thanks to the spectral decomposition of the adjacency matrix, which reads as $A=B\Lambda^{\left(A\right)}B^{T}$ where $\Lambda^{\left(A\right)}=\mathrm{diag}\left\{\lambda_{1}^{\left(A\right)},\ldots ,\lambda_{\left|V\right|}^{\left(A\right)}\right\}$ is a diagonal matrix containing the eigenvalues in increasing order and B contains the corresponding unitary-length eigenvectors, thus:
  (A15) $$SC\left(v_{i}\right)=\sum_{j=1}^{\left|V\right|}e^{\lambda_{j}^{\left(A\right)}}{\left(b_{j}\left(v_{i}\right)\right)}^{2},$$
  where $\lambda_{j}$ and $b_{j}$ are the eigenvalue and eigenvector associated to node $v_{j}\in V$ and $b_{j}\left(v_{i}\right)$ indicates the value related to $v_{i}$ in the *j*^th^ eigenvector;
- The Estrada Index [116] EI(G) of a graph G quantifies the compactness (or ‘folding’, since the Estrada Index was indeed originally proposed in order to study molecular 3D compactness) of a graph starting from the spectral decomposition of the adjacency matrix (as in the subgraph centrality):
  (A16) $$EI\left(G\right)=\sum_{j=1}^{\left|V\right|}e^{\lambda_{j}^{\left(A\right)}};$$
- The harmonic centrality [117] $HC(v_{i})$ is the sum of inverse shortest paths distances from a given node $v_{i}\in V$ to all other nodes:
  (A17) $$HC\left(v_{i}\right)=\sum_{\begin{array}{c} j=1\\ j\neq i \end{array}}^{\left|V\right|}\frac{1}{\delta (v_{i},v_{j})};$$
- The global reaching centrality [118] GRC(G) of a graph G is the average (over all nodes) of the difference between the maximum local reaching centrality and each node’s local reaching centrality. Formally:
  (A18) $$GRC\left(G\right)=\frac{\sum_{i=1}^{\left|V\right|}\left(LRC_{\max}-LRC\left(v_{i}\right)\right)}{|V|-1},$$
  where $LRC(v_{i})$ is the local reaching centrality of node $v_{i}\in V$ and $LRC_{\max}$ is the maximum local reaching centrality amongst all nodes. In turn, the local reaching centrality for a given node $v_{i}$ is defined as the percentage of nodes reachable from $v_{i}$;
- The average clustering coefficient [119] ACC(G) of a graph G is given by:
  (A19) $$ACC\left(G\right)=\frac{1}{\left|V\right|}\sum_{i=1}^{\left|V\right|}cc\left(v_{i}\right),$$
  where $cc(v_{i})$ is the clustering coefficient for node $v_{i}$, defined as:
  (A20) $$cc\left(v_{i}\right)=\frac{2\cdot tri(v_{i})}{DC\left(v_{i}\right)\cdot \left(DC(v_{i})-1\right)},$$
  where, in turn, $tri(v_{i})$ is the number of triangles passing through node $v_{i}$ and $D(v_{i})$ is its degree;
- The average neighbour degree [120] $AND(v_{i})$ of node $v_{i}\in V$ is given by:
  (A21) $$AND\left(v_{i}\right)=\frac{1}{\left|N(v_{i})\right|}\sum_{v_{j}\in N\left(v_{i}\right)}D\left(v_{j}\right),$$
  where $N(v_{i})$ is the set of neighbours of node $v_{i}$.

Apart from ACC, EI and GRC, which are global characteristics (i.e., related to the whole graph), the others are local characteristics (i.e., related to each node or edge). As such, it is impossible to compare graphs having different sizes (number of nodes and/or edges) by considering their local centralities. The second representation $X^{\left(2\right)}$ sees each protein as a 27-length real-valued vector containing $\bar{DC}$, $\tilde{DC}$, $\bar{e}$, $\tilde{e}$, $\bar{p}$, $\tilde{p}$, $\bar{k}$, $\tilde{k}$, $\bar{CC}$, $\tilde{CC}$, $\bar{BC}$, $\tilde{BC}$, $\bar{EBC}$, $\tilde{EBC}$, $\bar{LC}$, $\tilde{LC}$, $\bar{ELC}$, $\tilde{ELC}$, $\bar{SC}$, $\tilde{SC}$, EI, $\bar{HC}$, $\tilde{HC}$, GRC, ACC, $\bar{AND}$, $\tilde{AND}$ (where bar and tilde indicate the average and standard deviation centrality across nodes/edges).

### Appendix A.3. Energy and Laplacian Energy

Let G=(V,E) be a graph and let V and E be the set of nodes and edges, respectively. Since in this work unweighted and undirected graphs are considered, the adjacency matrix A is a binary |V|×|V| matrix defined as:

(A22) $$A_{i,j}=\left\{\begin{array}{cc} 1 & \;\mathrm{if}\;(v_{i},v_{j})\in E\\ 0 & \;\mathrm{otherwise}. \end{array}\right.$$

From A, it is possible to define the diagonal |V|×|V| degree matrix as:

(A23) $$D_{i,j}=\left\{\begin{array}{cc} D(i) & \;\mathrm{if}\;i=j\\ 0 & \;\mathrm{otherwise}, \end{array}\right.$$

where D(i) is the degree of the *i*th node. In turn, from A and D, it is possible to define the Laplacian matrix as:

(A24) L=D−A,

The spectrum and Laplacian spectrum of G are defined as the set of eigenvalues from A and L, respectively [121]:

(A25) $$\lambda^{\left(A\right)}=\left\{\lambda_{1}^{\left(A\right)},\ldots ,\lambda_{\left|V\right|}^{\left(A\right)}\right\},$$

(A26) $$\lambda^{\left(L\right)}=\left\{\lambda_{1}^{\left(L\right)},\ldots ,\lambda_{\left|V\right|}^{\left(L\right)}\right\}.$$

From Equations (A25) and (A26), it is possible to define the graph energy *E* and the Laplacian energy LE as

(A27) $$E=\sum_{i=1}^{\left|V\right|}\left|\lambda_{i}^{\left(A\right)}\right|,$$

(A28) $$LE=\sum_{i=1}^{\left|V\right|}\left|\lambda_{i}^{\left(L\right)}-\frac{2|E|}{\left|V\right|}\right|.$$

The third representation $X^{\left(3\right)}$ sees each protein as a 2-length real-valued vector containing *E* and LE.

### Appendix A.4. Nodes Functional Cartography

Guimerà and Amaral in their seminal work [122] proposed a methodology in order to extract functional modules from a graph by maximising its modularity using simulated annealing [123]. Their definition of modularity takes into account both within-module degree and between-module degree with the idea that a good graph partition (i.e., high modularity) must have many within-module links and few between-module links.

Each node is then assigned with two scores: the *z*-score and the participation coefficient *P*. The former measures how well-connected a given node is with respect to other nodes in its own module. The latter quantifies how many connections a given nodes has with respect to nodes belonging to different modules.

The z−P plane has been heuristically divided into seven regions and each node can be classified into one of seven functional roles by considering its *z*-score and its participation coefficient *P*. Nodes having z<2.5 are non-hubs, whereas nodes having z≥2.5 are hubs. In turn, non-hub nodes can be divided in: ultra-peripherals (if P≤0.05), peripherals (if P∈(0.05,0.62]), non-hub connectors (if P∈(0.62,0.8]) and non-hub kinless (if P>0.8). Finally, hub nodes can be divided in: provincial hubs (if P≤0.3), connector hubs (if P∈(0.3,0.75]) and kinless hubs (if P>0.75).

The fourth representation $X^{\left(4\right)}$ sees each protein as an 8-length real-valued vector containing the modularity (as returned by the simulated annealing) and the percentage of nodes belonging to each functional role.

### Appendix A.5. Heat Content Invariant

From the graph Laplacian and degree matrices (Equations (A24) and (A23), respectively), the normalised Laplacian matrix can be evaluated as:

(A29) $$\tilde{L}=D^{-\frac{1}{2}}LD^{-\frac{1}{2}},$$

The spectral decomposition of $\tilde{L}$ reads as:

(A30) $$\tilde{L}=V\Lambda V^{T},$$

where $\Lambda =\mathrm{diag}\left\{\lambda_{1}^{\left(\tilde{L}\right)},\ldots ,\lambda_{\left|V\right|}^{\left(\tilde{L}\right)}\right\}$ is a diagonal matrix containing the eigenvalues in increasing order and V contains the corresponding unitary-length eigenvectors.

The heat equation associated to $\tilde{L}$ is given by [124,125]:

(A31) $$\frac{\partial H(t)}{\partial t}=-\tilde{L}H\left(t\right),$$

where H(t) is the |V|×|V| heat kernel matrix at time *t*. The heat content HC(t) of H(t) is given by:

(A32) $$\begin{array}{cc} HC(t) & =\sum_{v_{i}\in V}\sum_{v_{j}\in V}H_{i,j}\left(t\right)\\ \; & =\sum_{v_{i}\in V}\sum_{v_{j}\in V}\sum_{k=1}^{\left|V\right|}\exp\left\{-\lambda_{k}^{\left(\tilde{L}\right)}t\right\}v_{k}\left(v_{i}\right)v_{k}\left(v_{j}\right), \end{array}$$

where $v_{k}\left(v_{i}\right)$ is the value related to node $v_{i}$ in the *k*th eigenvector.

The MacLaurin series for the negative exponential reads as:

(A33) $$\exp\left\{-\lambda_{k}^{\left(\tilde{L}\right)}t\right\}=\sum_{m=0}^{\infty }\frac{{\left(-\lambda_{k}^{\left(\tilde{L}\right)}t\right)}^{m}t^{m}}{m!}$$

and substituting Equation (A33) in Equation (A32) yields:

(A34) $$HC\left(t\right)=\sum_{v_{i}\in V}\sum_{v_{j}\in V}\sum_{k=1}^{\left|V\right|}\sum_{m=0}^{\infty }\frac{{\left(-\lambda_{k}^{\left(\tilde{L}\right)}t\right)}^{m}t^{m}}{m!}v_{k}\left(v_{i}\right)v_{k}\left(v_{j}\right)$$

By re-writing Equation (A32) in terms of power series as:

(A35) $$HC\left(t\right)=\sum_{m=0}^{\infty }q_{m}t^{m},$$

where the set of coefficients $q_{m}$ are the so-called heat content invariants and can be evaluated in closed-form as:

(A36) $$q_{m}=\sum_{i=1}^{\left|V\right|}\left({\left(\sum_{v\in V}v_{i}\left(v\right)\right)}^{2}\right)\frac{{\left(-\lambda_{i}^{\left(\tilde{L}\right)}\right)}^{m}}{m!}.$$

The fifth representation $X^{\left(5\right)}$ sees each protein as a 4-length real-valued vector containing the first four coefficients from Equation (A36); that is $q_{1},q_{2},q_{3},q_{4}$.

### Appendix A.6. Heat Kernel Trace

Recalling the heat equation from Equation (A31) and the spectral decomposition of the normalised Laplacian matrix from Equation (A30), the solution to the former (already in Equation (A32)) reads as:

(A37) $$\begin{array}{cc} H(t) & =\exp\left\{-t\tilde{L}\right\}=V\exp\left\{-t\Lambda \right\}V^{T}\\ \; & =\sum_{i=1}^{\left|V\right|}\exp\left\{-\lambda_{i}^{\left(\tilde{L}\right)}t\right\}v_{i}v_{i}^{T}. \end{array}$$

The heat kernel trace is evaluated by taking the trace of H(t):

(A38) $$HT\left(t\right)=\mathrm{Tr}\left\{H(t)\right\}=\sum_{i=1}^{\left|V\right|}\exp\left\{-\lambda_{i}^{\left(\tilde{L}\right)}t\right\}.$$

The sixth representation $X^{\left(6\right)}$ sees each protein as a 10-length real-valued vector containing the heat kernel trace for t=1,2,…,10. These values for *t* have been chosen by visual inspection: indeed, for t>10 the heat kernel trace decay makes proteins undistinguishable one another.

### Appendix A.7. Size

The seventh representation $X^{\left(7\right)}$ sees each protein as a 4-length real-valued vector containing the number of nodes, the number of edges, the number of protein chains and the radius of gyration. Whilst the first two items are rather straightforward, the latter two items deserve some further comments. Proteins are composed by one or more amino-acids chains (linear polymers), thus the number of chains may impact on the overall protein size. Finally, the radius of gyration [126] is a measure of how-compact is the overall folded protein structure with respect to its centre of mass.

### Appendix A.8. Normalised Laplacian Spectral Density

Recalling the spectral decomposition of the normalised Laplacian matrix from Equation (A30), let $\lambda^{\left(\tilde{L}\right)}=\left\{\lambda_{1}^{\left(\tilde{L}\right)},\ldots ,\lambda_{\left|V\right|}^{\left(\tilde{L}\right)}\right\}$ be the normalised Laplacian spectrum (namely, the set of eigenvalues from $\tilde{L}$). One of the interesting properties of the normalised Laplacian matrix is that its spectrum lies in range [0,2], regardless of the underlying graph [127]. The size of the spectrum, however, equals the number of nodes and therefore one cannot easily compare graphs having different sizes just by considering their respective spectra. In order to overcome this problem, following previous works [45,50], it is possible to estimate the (normalised Laplacian) spectral density using a kernel density estimator (also known as Parzen window [128]) equipped with the Gaussian kernel. The spectral density thus has the form:

(A39) $$p\left(x\right)=\frac{1}{\left|V\right|}\sum_{i=1}^{\left|V\right|}\frac{1}{\sqrt{2\pi \sigma^{2}}}\cdot \exp\left\{\frac{-{\left(x-\lambda_{i}^{\left(\tilde{L}\right)}\right)}^{2}}{2\sigma^{2}}\right\},$$

where σ is the kernel bandwidth which determines the estimate resolution. Following [50], the Scott’s rule [129] has been used in order to determine the proper bandwidth value, hence:

(A40) $$\sigma =\frac{3.5\cdot \mathrm{std}\left\{\lambda^{\left(\tilde{L}\right)}\right\}}{{\left|\lambda^{\left(\tilde{L}\right)}\right|}^{1/3}}.$$

In this manner, the bandwidth scales in a graph-wise fashion by considering each graph’s spectrum size (denominator) and its standard deviation (numerator). Let $G_{1}$ and $G_{2}$ be two graphs, their distance can be evaluated by considering the $\ell_{2}$ norm between their respective spectral densities $p_{1}\left(x\right)$ and $p_{2}\left(x\right)$:

(A41) $$d\left(G_{1},G_{2}\right)=\int_{0}^{2}{\left(p_{1}\left(x\right)-p_{2}\left(x\right)\right)}^{2}dx.$$

The same operation can be carried in the discrete domain by extracting *n* samples from p(x) (equal for all graphs) and the latter collapses into the standard Euclidean distance.

The eighth representation $X^{\left(8\right)}$ sees each protein as an 100-length real-valued vector containing n=100 samples uniformly drawn from their respective normalised Laplacian spectral densities.

## Appendix B. Selected Prototypes

In the following, the sets of proteins elected as prototypes for each of the seven classification problems are shown. In order to shrink the output size, our a posteriori analysis has been carried only on proteins which have been selected in all of the five runs of the genetic algorithm (in order to remove ’spurious’ representatives due to randomness in the optimisation procedure).

**Table A1:** Selected proteins in order to discriminate EC 1 (oxidoreductases) vs. all the rest.

PDB ID	Notes/Description
1KOF	Transferase
1XFG	Transferase
3E2R	Oxydoreductase
4TS9	Transferase
1ZDM	Signalling Protein
1MPG	Hydrolase
1QQQ	Transferase

**Table A2:** Selected proteins in order to discriminate EC 2 (transferases) vs. all the rest.

PDB ID	Notes/Description
3EDC	LAC repressor (signalling protein)
1DKL	Hydrolase
1JKJ	Ligase
2DBI	Unknown function
3UCS	Chaperone
1LX7	Transferase
2GAR	Transferase
3ILI	Transferase
1S08	Transferase
4IXM	Hydrolase
4XTJ	Isomerase
1KW1	Lyase
1BDH	Transcription factor (DNA-binding)
4PC3	Elongation factor (RNA-binding)
5G1L	Isomerase

**Table A3:** Selected proteins in order to discriminate EC 3 (hydrolases) vs. all the rest.

PDB ID	Notes/Description
4RZS	Transcription factor (signalling protein)
1ZDM	Signalling protein
3I7R	Lyase
1HW5	Signalling protein
1SO5	Lyase

**Table A4:** Selected proteins in order to discriminate EC 4 (lyases) vs. all the rest.

PDB ID	Notes/Description
2BWX	Hydrolase
3UWM	Oxydoreductase
2H71	Electron transport
1D7A	Lyase
4DAP	DNA-binding
1SPV	Structural genomics, unknown function
1EXD	Ligase + RNA-binding
1X83	Isomerase
3ILJ	Transferase
2D4U	Signalling protein
1JNW	Oxydoreductase
1TRE	Oxydoreductase
1ZPT	Oxydoreductase
3LGU	Hydrolase
1IB6	Oxydoreductase
3C0U	Structural genomics, unknown function
5GT2	Oxydoreductase
2RN2	Hydrolase
4L4Z	Transcription regulator
3CMR	Hydrolase
1NQF	Transport protein
1GPQ	Hydrolase
4ODM	Isomerase + chaperone
2NPG	Transport protein
2UAG	Ligase
1OVG	Transferase
3AVU	Transferase
1RBV	Hydrolase
5AB1	Cell adhesion
1TMM	Transferase
4NIY	Hydrolase
4WR3	Isomerase

**Table A5:** Selected proteins in order to discriminate EC 5 (isomerases) vs. all the rest.

PDB ID	Notes/Description
4ITX	Lyase
2BWW	Hydrolase
5IU6	Transferase
1ODD	Gene regulatory
5G5G	Oxydoreductase
1G7X	Transferase
2E0Y	Transferase
2SCU	Ligase
1HO4	Hydrolase
3RGM	Transport Protein
1OAC	Oxydoreductase
5MUC	Oxydoreductase
3OGD	Hydrolase + DNA binding
4K34	Membrane protein
1Q0L	Oxydoreductase
1G58	Isomerase
5M3B	Transport protein
2WOH	Oxydoreductase
2PJP	Translation regulation (RNA-binding)

**Table A6:** Selected proteins in order to discriminate EC 6 (ligases) vs. all the rest.

PDB ID	Notes/Description
2OLQ	Lyase
1JDI	Isomerase
4NIG	Oxydoreductase + DNA-binding
5T03	Transferase
5FNN	Oxydoreductase
2Z9D	Oxydoreductase
2V3Z	Hydrolase
4ARI	Ligase + RNA-binding
3LBS	Transport protein
4QGS	Oxydoreductase
5B7F	Oxydoreductase
2ABH	Transferase

**Table A7:** Selected proteins in order to discriminate not-enzymes vs. all the rest.

PDB ID	Notes/Description
1SPA	Transferase
2YH9	Membrane protein
1NQF	Transport protein
1LDI	Transport protein
1TIK	Hydrolase
1MWI	Hydrolase + DNA-binding
1GEW	Transferase
5CKH	Hydrolase
3ABQ	Lyase
3B6M	Oxydoreductase
